# Confirmation and inheritance of glufosinate resistance in an *Amaranthus palmeri* population from North Carolina

**DOI:** 10.1002/pei3.10154

**Published:** 2024-06-25

**Authors:** Eric A. L. Jones, Jeffrey C. Dunne, Charles W. Cahoon, Katherine M. Jennings, Ramon G. Leon, Wesley J. Everman

**Affiliations:** ^1^ Department of Crop and Soil Sciences North Carolina State University Raleigh North Carolina USA; ^2^ Department of Horticultural Science North Carolina State University Raleigh North Carolina USA; ^3^ Current Position and Address: Department of Agronomy, Horticulture, and Plant Science South Dakota State University Brookings South Dakota USA

**Keywords:** glufosinate, herbicide resistance, inheritance, oligogenic

## Abstract

A putative glufosinate‐resistant *Amaranthus palmeri* population was reported in 2015 in Anson County, North Carolina. The results from dose–response assays conducted in the field suggested plants were surviving lethal rates of glufosinate. Dose–response assays conducted in the glasshouse determined the Anson County accession exhibited reduced susceptibility to glufosinate compared to three glufosinate‐susceptible populations. The LD_50_ values (210–316 g ai ha^−1^) for the Anson County population were always higher than the LD_50_ values (118–158 g ai ha^−1^) for the tested susceptible populations from the dose–response assays. Anson County plants that survived lethal glufosinate rates were reciprocally crossed with susceptible plants to create F_1_ genotypes and treated with a lethal rate of glufosinate (267 g ai ha^−1^; ascertained from glasshouse dose–response assay) to determine the distribution of injury and survival for each cross compared to a cross of susceptible parents. The distribution of injury was non‐normal for the crosses containing an Anson County plant compared to the cross with a susceptible parent. Survival was 68%–84% for crosses containing an Anson County plant, whereas the survival was significantly reduced to 35% for the susceptible plant cross. Chi‐square goodness of fit tests were used to test inheritance models to describe the responses of the genotypes. The resistant × susceptible crosses were best described with a heterozygous two loci with incomplete dominance model compared to the resistant × resistant cross that was best described with a heterozygous single locus with incomplete dominance model. The Anson County population has evolved resistance to glufosinate that is heritable and likely conferred by an oligogenic mechanism with incomplete dominance.

## 1 INTRODUCTION

*Amaranthus palmeri* S. Watson (Palmer amaranth) is one of the most pervasive and troublesome weeds in field crops in the United States (Van Wychen, 2020; Webster & Nichols, 2012). *Amaranthus palmeri* is difficult to control due to prolonged emergence, rapid vegetative growth, and high fecundity (Keeley et al., 1987; Mahoney et al., 2021; Sellers et al., 2003). The species has evolved resistance to nine unique herbicide sites of action and multiple herbicide‐resistant populations are common, contributing to the problem (Jhala et al., 2014; Kohrt et al., 2017; Shyam et al., 2021). Widespread evolution of acetolactate synthase (ALS [EC 2.2.1.6]; herbicide group [HG] 2)‐ and 5‐enolpyruvylshikimate‐3‐phosphate synthase (EPSPS [EC 2.5.1.19]; HG 9) (e.g., glyphosate)‐inhibiting herbicide‐resistant *A. palmeri* in North Carolina has resulted in reliance on other herbicides for control (Cahoon et al., 2015; Mahoney et al., 2020). Glufosinate has been relied on extensively in North Carolina cotton as transgenic varieties were available earlier than glufosinate‐tolerant soybean and provided a postemergence option for ALS‐inhibiting herbicide‐ and glyphosate‐resistant weeds (Culpepper & York, 1999; Duke, 2014; Everman et al., 2007). Glufosinate is a non‐selective, fast‐acting contact herbicide that inhibits glutamine synthetase (GS [EC 6.3.1.2]; HG 10) resulting in the production of reactive oxygen species that disrupt cell membrane integrity (Takano et al., 2019).

Glufosinate is efficacious on many weed species that inhabit row crops (Corbett et al., 2004; Hoss et al., 2003). Although glufosinate‐resistant *A. palmeri* has not previously been confirmed in North Carolina, the overreliance on glufosinate for weed control in the Midsouth United States has led to the evolution of several glufosinate‐resistant *A. palmeri* populations (Carvalho‐Moore et al., 2022; Noguera et al., 2022; Priess et al., 2022). Although glufosinate‐resistant *A. palmeri* is not common, control failures are common due to applying the herbicide to large weeds or under adverse environmental conditions (Coetzer et al., 2001; Sellers et al., 2004). Farmers tend to notice control failures attributable to herbicide resistance when approximately 35% of plants in the field population have evolved resistance and the susceptibility to the herbicide is reduced by 2‐ to 4‐fold (Gressel & Segel, 1990; Squires et al., 2021). Glufosinate control failures on an ALS‐inhibiting herbicide‐ and glyphosate‐resistant *A. palmeri* population were noticed by a farmer in Anson County, North Carolina after the first season of spraying glufosinate (450 g ai [active ingredient] ha^−1^) in glufosinate‐tolerant cotton. Interestingly, as signified by a recently conducted survey, glufosinate control failures were evident in Piedmont (Anson County) cotton compared to other crops and regions of North Carolina (Jones, Cahoon, et al., 2022).

Since glufosinate control failures can occur via adverse environmental conditions and applications to large plants, *A. palmeri* plants recurrently exposed to sublethal rates of glufosinate could accumulate allele(s) that facilitate resistance as demonstrated by previous research (Matzrafi et al., 2020; Neve & Powles, 2005; Tehranchian et al., 2017). Understanding whether the inheritance of these resistance gene(s) are Mendelian or maternal would be important to determine what phenotypes the offspring would exhibit after being treated with glufosinate (Jasienuiek et al., 1996). The other important aspect for target‐site glufosinate resistance is there are two isoforms of GS: cytoplasmic and chloroplastic; therefore, the inheritance may be dependent on the mechanism of resistance (Avila‐Garcia et al., 2012; Hirel & Gadal, 1981). Carvalho‐Moore et al. (2022) demonstrated that one of the glufosinate‐resistant *A. palmeri* populations had increased copy number of *GS,* but the resistance mechanism(s) of the other resistant populations were different and yet to be determined. Thus, the mechanism(s) of resistance can differ among populations (Délye et al., 2013; Gaines et al., 2020). The objectives of this research were (1) to determine whether the Anson County *A. palmeri* population has evolved resistance to glufosinate and (2) propose an inheritance model for the resistance trait(s).

## 2 MATERIALS AND METHODS

### 2.1 Field dose–response and sequential applications

Field experiments were established at the farmer's field in Anson County in 2015. All weeds were controlled with paraquat (560 g ai ha^−1^) prior to experiment establishment using a CO_2_‐powered backpack sprayer with 11002XR (TeeJet Technologies, Wheaton, IL, USA) spray nozzles at an output of 140 L ha^−1^ at 241 kPa with a speed of 4.8 kilometers h^−1^ and 46 cm above the target weed height. The experimental design was a randomized complete block with four replications. Individual plots were 3 × 3 m. Glufosinate (Liberty 280 SL, BASF, Research Triangle Park, NC) was applied at eight rates ranging from 590 to 2360 g ai ha^−1^ to 8 cm tall (6‐leaf) plants using the application methodology as described above prior to each experiment establishment (Table 1). In addition, treatments of sequential applications (660 followed by (fb) 660, 880 fb 880, and 880 fb 660 g ai ha^−1^) of glufosinate were applied in the same experiment as well; applications were made 7 days apart. The sequential applications were tested to mimic applications a farmer might make if control failure occurred (Vann et al., 2017). The experiments also included a non‐treated control. A visual rating scale ranging from 0% to 100% was used to assess control 14 days after treatment (DAT); where 0% equaled no control and 100% equaled complete control. The experiment was conducted twice within the field where control failure was reported; where each experimental run was separated in time (21 days) and conducted on different *A. palmeri* cohorts separated by germination. Approximately 48 *Amaranthus palmeri* plants that survived glufosinate (875 g ai ha^−1^) were transplanted into pots containing the field soil, maintained in a glasshouse, allowed to mature and produce seed. The plants were open pollinated, and seeds were hand‐threshed and stored at 0 C separately for four selected female plants. Four selected female plants (A1, A2, A3, and A4) produced enough for research continuation.

**TABLE 1:** Glufosinate rates used in the field dose–response, Anson County accession dose–response under glasshouse conditions, and the F_1_ plants dose–response compared to populations with different glufosinate exposure dose–response experiments.

Experiment	Glufosinate rates
	g ai ha^−1^
*Field dose–response*	0, 590, 650, 875, 1189, 1302, 1465, 1750, 2340
*Anson County accession dose–response*	0, 10, 20, 40, 60, 90, 120, 180, 240, 475, 715, 955, 1190
*F*_*1*_ *plants dose–response*	0, 80, 163, 202, 245, 327, 450, 900

### 2.2 Anson County accession dose–response under glasshouse conditions

Seeds from each Anson County female accession (A1, A2, A3, and A4) surviving field‐applied glufosinate (875 g ai ha^−1^) were sown into separate 21 cm by 28 cm flats containing potting soil (Sunshine Mix #2, Sungro Horticulture, Agawam, MA, USA). A known glufosinate‐susceptible *A. palmeri* population collected from a North Carolina State University research farm in Johnston County, North Carolina (35.66 N, 78.50 W) in 2013 was included in the experiment (Poirier et al., 2014). Plants were maintained in the glasshouse at 31/24 C diurnal fluctuations and overhead irrigated to maintain the soil at field capacity. Sunlight was supplemented with 600–1000 μmol m^−2^ s^−1^ PPFD of artificial light set to a 14‐hr photoperiod. Two plants were then transplanted when approximately 2 cm in height to 10‐cm pots containing the same potting soil. The resulting experimental unit was two plants pot^−1^. Glufosinate was applied to *A. palmeri* plants when 7.6 to 10 cm in height (4‐ to 6‐leaf) with a CO_2_‐powered backpack sprayer with 11002XR spray nozzles calibrated to deliver 140 L ha^−1^ at 241 kPa with a speed of 4.8 kilometers h^−1^ and 46 cm above the target weed height. Glufosinate was applied at twelve rates ranging from 10 to 1190 g ai ha^−1^ (Table 1). The experiment included a non‐treated control for each accession. Treatments were arranged in a randomized complete block design with five replications and the experiment was conducted twice, resulting in 120 plants sprayed per accession. Plant survival was recorded on a binomial scale where 0 equaled plant death (no green vegetative tissue) and 1 equaled plant survival (visible green vegetative tissue) 21 DAT. After the plant survival evaluation, both plants were harvested and dried for 72 h at 60°C then weighed to determine aboveground dry biomass.

### 2.3 A4 plants dose–response compared to populations with different glufosinate exposure

Two glufosinate‐susceptible *A. palmeri* populations (Edgecombe [35.89 N, 77.68 W] and Lenoir County [35.29 N, 77.65 W], North Carolina) were collected in 2020 and 2019 from soybean fields on North Carolina State University research farms, respectively and handled as described above. These two *A. palmeri* populations were selected based on glufosinate use history (Edgecombe County: high use, ~20 years of glufosinate applications; Lenoir County: low use, ~5 years of glufosinate applications) (WJE, personal communication). Seeds from the A4 accession, Edgecombe County, and Lenoir County populations were sown and curated as described above. Five plants were transplanted when approximately 2 cm in height to 15‐cm pots containing the same potting mix combined with sand to achieve a 4:1 w w‐^1^ ratio and 1 g of Osmocote Flower Food Granules (14–14‐14) (The Scotts Company LLC, Marysville, OH, USA). The resulting experimental unit was five plants pot^−1^. Glufosinate was applied at seven rates ranging from 81 to 900 g ai ha^−1^ to plants 7.6 to 10 cm in height (4‐ to 6‐leaf) with the application methodology as described above (Table 1). A non‐treated control per population was included in the experiment. Treatments were arranged in a completely randomized design with four replications and the experiment was conducted twice separated in time by 7 days, resulting in 80 plants tested per population. Plant survival was evaluated 21 DAT as described above. Biomass data was not collected from this experiment to ensure there were ample plants for making biparental crosses and seed production.

### 2.4 F_1_ genotype resistance inheritance

Plants from the dose–response study described above were collected for conducting biparental crosses to determine whether glufosinate resistance was a heritable trait and the number of gene(s) responsible for the resistance. An A4 male surviving 450 g glufosinate ha^−1^ (RM) and female (RF_1_) plant surviving 267 g glufosinate ha^−1^ were crossed as well as a RM and an A4 female plant surviving glufosinate (450 g ai ha^−1^) (RF_2_) were crossed with non‐treated Lenoir County male (SM) and female (SF) plants (Figure S1). These crosses were made to determine if the resistance mechanism was controlled by a single gene and if the resistance gene(s) were maternally or Mendelian inherited. Plants were transplanted into 38‐cm pots containing the potting media and 5 g of pellet fertilizer as described above after the dose–response evaluation period. Plants were allowed to acclimate until 7 days after transplanting and artificial photoperiods (8 h day/16 h night) were implemented to induce flowering (Sauer, 1957). The artificial photoperiod was induced by placing a PVC structure covered with black, opaque plastic over the pots containing the plants. Flowering initiated approximately 7–14 days after inducing the artificial photoperiod. At this point, paired plants of each cross were covered with a pollination bag for crossing and to avoid exposure to pollen from other plants and removed from the artificial photoperiod treatment. The pollination bag for each cross was manually shaken daily to ensure pollen movement. Flowering plants remained in the pollination bags for approximately 1 month. Female plants were then harvested and dried in paper bags at 10–25°C for approximately 2 weeks. Plants were then threshed by hand and cleaned with an air column separator (South Dakota Seed Blower [Seedburo Equipment Company, Chicago, IL, USA]). The seeds from each cross were placed into a petri dish with a small amount of de‐ionized water and wet‐chilled at 6°C for 2 weeks to break dormancy. The petri dishes without lids were then placed in a dryer at 65°C for 48 h to reduce their moisture content before storage (Leon et al., 2006).

Seeds from each biparental cross were sown and established as described above. Plants were transplanted to 10‐cm pots when reaching approximately 7 cm in height. Ten plants (genotypes) were arbitrarily selected from each biparental cross; resulting in 40 genotypes tested. Each of the genotypes (plants) were cloned three times per experimental run to control for genetic variation across the experimental runs (environment). Clones were produced by cutting axillary stems from the plants, submerging them in water for approximately 30 s to prevent xylem cavitation, dipping in a rooting hormone (indole‐3‐butyric acid [Garden Safe Take‐Root, Spectrum Brands, Incorporated, Middleton, WI]) and transplanted into a separate 10‐cm pots. Three experimental runs were conducted to represent different environments for each of the original 30 clones tested per biparental cross. Glufosinate was applied to 7.6–10 cm (4‐ to 6‐leaf) plants at a discriminating rate (267 g ai ha^−1^) identified from dose–response studies using the application methodology described above. Percent injury was visually estimated using a rating scale ranging from 0% to 100%; where 0% equaled no injury and 100% equaled plant death. Percent was then categorized into resistant (<70%), intermediate resistant (>70 to 90%), and susceptible (>90%). Plant survival was recorded 21 DAT as described above.

### 2.5 Statistical analysis

#### 2.5.1 Field dose–response and sequential application experiments

Plant control was modeled using a three‐parameter log‐logistic equation using Sigmaplot 14.0 (Systat Software, Palo Alto, CA, USA).

(1) $$y=a/\left[1+\left(x/x0\right)b\right],$$

where *a* is the upper asymptote, *x* is the herbicide rate, *x0* equals the ED_50_ (effective dose to visually control 50% of the population) rate, and *b* is the slope at *x0*. The ED_50_ for each *A. palmeri* cohort was derived from the regression equation.

Control data from the sequential applications were analyzed separately and subjected to ANOVA using PROC GLIMMIX in SAS v. 9.4 (Statistical Analysis Software, Cary, NC) (*α* ≤ 0.05), where sequential application and cohort were considered fixed effects, whereas replication was considered a random effect. Treatment means were separated using Fisher's LSD (*α* ≤ 0.05).

#### 2.5.2 Glasshouse dose–response experiments

Plant survival and biomass reduction were modeled using a three‐parameter log‐logistic model ((1)), where *a* is the upper asymptote, *x* is the herbicide rate, *x0* equals the LD_50_ (lethal dose to control 50% of the population) or GR_50_ (dose to reduce growth by 50%), and *b* is the slope at *x0*. The LD_50_/GR_50_ for each *A. palmeri* population was derived from the regression equations.

#### 2.5.3 Chi‐square goodness of fit

Injury and survival segregation patterns of the genotypes from each biparental cross were visualized using histograms, and genetic inheritance models exploring different expected segregation patterns were evaluated and tested with the chi‐square goodness‐of‐fit test (*α* = 0.05) using PROC FREQ in SAS 9.4. This test was used to evaluate the hypothesis that a single dominant gene was primarily responsible for glufosinate resistance. Since the Anson County plants (RM, SF_1_ and SF_2_) were not genotyped, the models used for the chi‐square were derived from assumptions based on the parental genotypes contributing to the resistance trait. An important assumption included in the models was the glufosinate‐susceptible parents (Lenoir County; SM and SF) were homozygous recessive at the resistance locus or loci. The parental genotypes considered were: single loci homozygous, single loci heterozygous, single loci heterozygous with incomplete dominance, two loci homozygous, two loci heterozygous, two loci homozygous for one allele and heterozygous for the other, and two loci heterozygous with incomplete dominance. The null hypotheses of the F_1_ genotypes from the biparental crosses failed to be rejected (*p* ≥ .05) when the number of surviving plants was not significantly different than the expected number of surviving plants and plant injury based on the observed ratios. Odds ratios were calculated by dividing the survival of the RM × SF and the RM × RF_1_ cross by the survival of the SM × RF_2_ cross. If the ratio was greater than 1, the resistance mechanism(s) was maternally inherited. If the ratio is less than 1, the resistance mechanism(s) was nuclear inherited.

#### 2.5.4 Heritability

Broad‐sense heritability was calculated for the F_1_ genotypes to determine the genetic variance that explains the phenotypic variance expressed by the clones from the biparental crosses based on the variances of injury and survival derived from analysis of variance (*p* ≤ .05). Broad‐sense heritability was calculated on a per plot and entry basis. Broad‐sense heritability on a per plot basis using the following equation:

(2) $$H^{2}=\frac{\text{σGenotype}}{\text{σGenotype}+\text{σGenotype}\times \text{Environment}+\text{σResidual}},$$

where *H* ^*2*^ equals broad‐sense heritability, *σ*Genotype equals genetic variance, *σ*Genotype × Environment equals the genetic by environment interaction, and *σ*Residual equals the residual error (population variance).

Broad‐sense heritability on a per entry basis was calculated using the following equation:

(3) H2=σGenotypeσGenotype+σGenotype×Environmentenvi+(σResidualenvi×reps,

where *H* ^*2*^ equals broad‐sense heritability, σGenotype equals genetic variance, σGenotype×Environment equals the genetic by environment interaction, *envi* equals the number of environments, σResidual equals the residual error (population variance) and *reps* equals the number of replications. Heritability was considered high, medium, or low, if values were >0.5, 0.5 to 0.25, and <0.25, respectively.

## 3 RESULTS

### 3.1 Field dose–response and sequential applications

**FIGURE 1:**
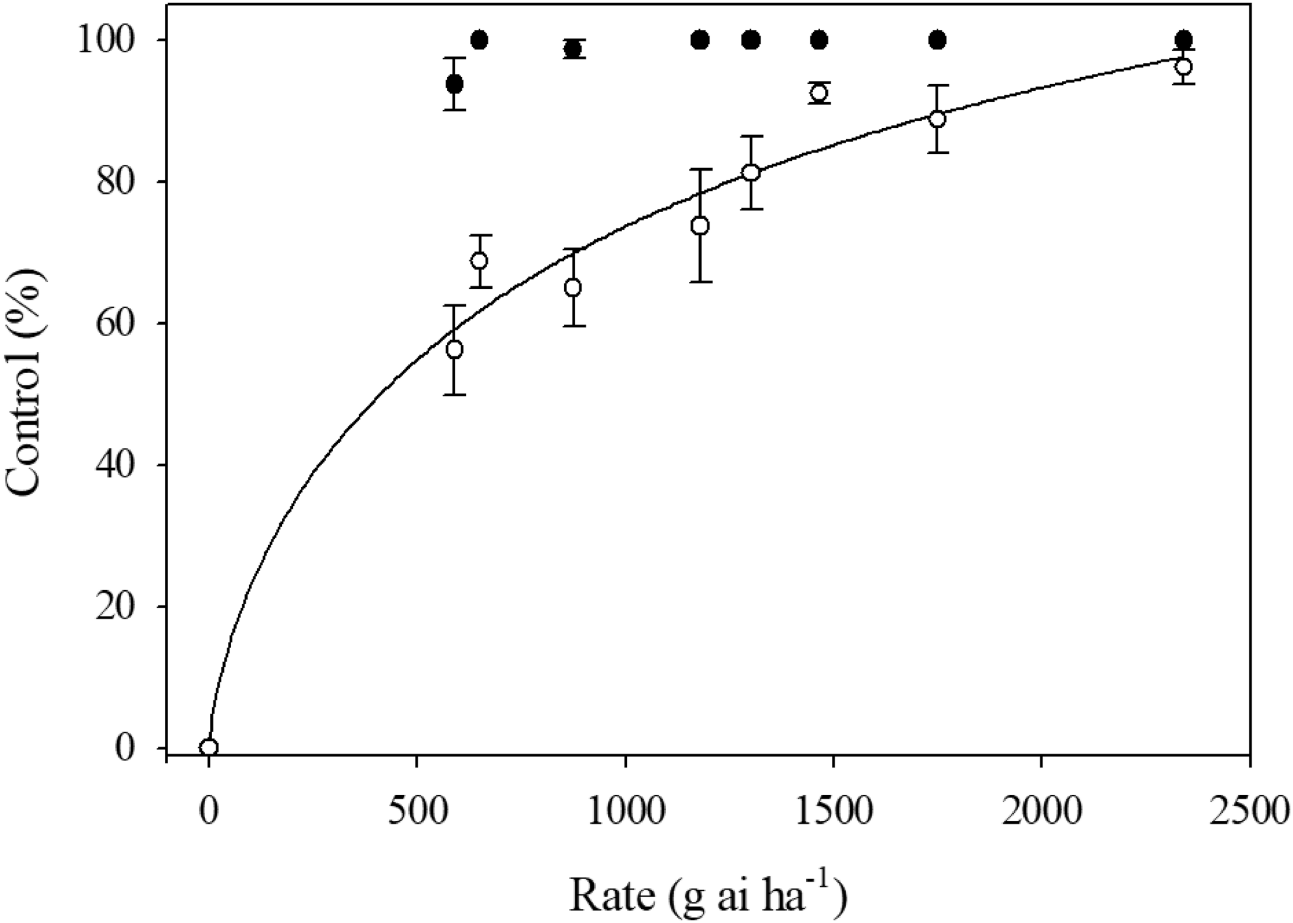
Dose–response curve fit to a three‐parameter logistic regression model for control of the Anson County *Amaranthus palmeri* population at the field site analyzed by cohort (black circle: cohort 1; white circle: cohort 2). The control of cohort 1 could not be modeled due to the high level of control incurred.

Control was variable for the two *A. palmeri* cohorts; thus, control data were analyzed by cohort (Figure 1). Control was greater than 90% for all glufosinate rates tested and control could not be modeled for the first cohort (Figure 1). Control was never greater than 70% with glufosinate rates lower than 875 g ai ha^−1^ for the second cohort, representing labeled and efficacious rates (Figure 1) (Anonymous, 2017). Control ranged from 74% to 96% with the higher rates (Figure 1). The ED_50_ for the second cohort was 410 g ai ha^−1^, which represents a historically lethal rate for *A. palmeri* (Coetzer et al., 2002; Corbett et al., 2004). Although the field dose–response experiment was not conducted at locations with glufosinate‐susceptible plants for comparison, the fact some plants survived singled applications of labeled glufosinate rates suggested that the Anson County population had reduced susceptibility to glufosinate. The sequential glufosinate applications controlled all the plants in both experimental runs (data not shown). This result suggests that resistance mechanism can be overwhelmed by applying greater than labeled rates (Gaines et al., 2020; Rigon et al., 2020).

### 3.2 Anson County accession dose–response under glasshouse conditions

**FIGURE 2:**
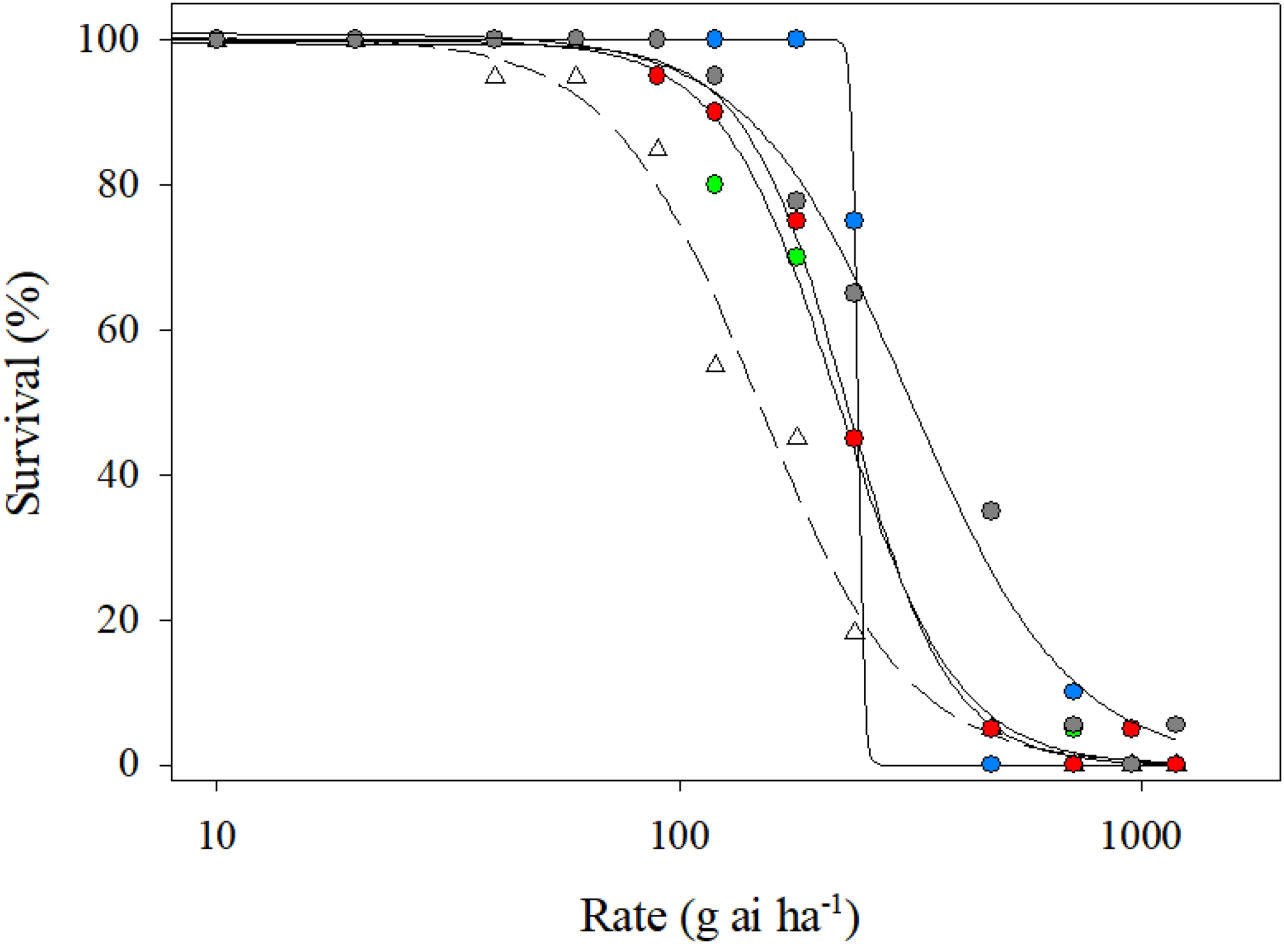
Dose–response curve fit to a three‐parameter logistic regression model for plant survival of the *Amaranthus palmeri* populations (putative resistant: Anson County; susceptible: Johnston County) treated with glufosinate. Error bars were removed for clarity. Legend: Anson County: filled circle [A1: blue; A2: red; A3: green; A4: gray]; Johnston County: open triangle.

*Survival*. The glufosinate LD_50_ values were 244, 231, 222, and 316 g ai ha^−1^ for the A1, A2, A3, and A4 accessions, respectively (Figure 2, Table 2). The LD_50_ was 149 g ai ha^−1^ for the Johnston County population; significantly lower than all the Anson County accessions (Figure 2, Table 2). The A4 accession exhibited the highest survival across the tested rates compared to the other accessions and the Johnston County population (Figure 2). The LD_50_ values exhibited by the Anson County accessions were similar to previously documented glufosinate‐resistant *A. palmeri* populations from Arkansas and Missouri, USA (Noguera et al., 2022; Priess et al., 2022).

**TABLE 2:** Parameter estimates from the three‐parameter logistic regression for plant survival of the putative glufosinate‐resistant Anson County *Amaranthus palmeri* accessions (A1, A2, A3, and A4) treated with glufosinate compared to the glufosinate‐susceptible Johnston County population.

Population/accession	Regression parameters^a^					
	*a*	*x*0	*b*	*r* ^2^	LD_50_	LD_90_
Johnston county	100.3	148.9	2.7	0.99	149	337
A1	100	243.8	69.5	0.99	244	244
A2	99.5	231.2	3.9	0.99	231	400
A3	99.9	221.7	3.4	0.99	222	418
A4	100.9	316.5	2.5	0.99	316	760

Abbreviations: LD_50_, lethal dose (g ai ha^−1^) to control 50% of the population; LD_90_, lethal dose (g ai ha^−1^) to control 90% of the population.

^a^ *a* is the upper asymptote, *x*0 equals the LD_50_, and *b* is the slope at *x*0.

**FIGURE 3:**
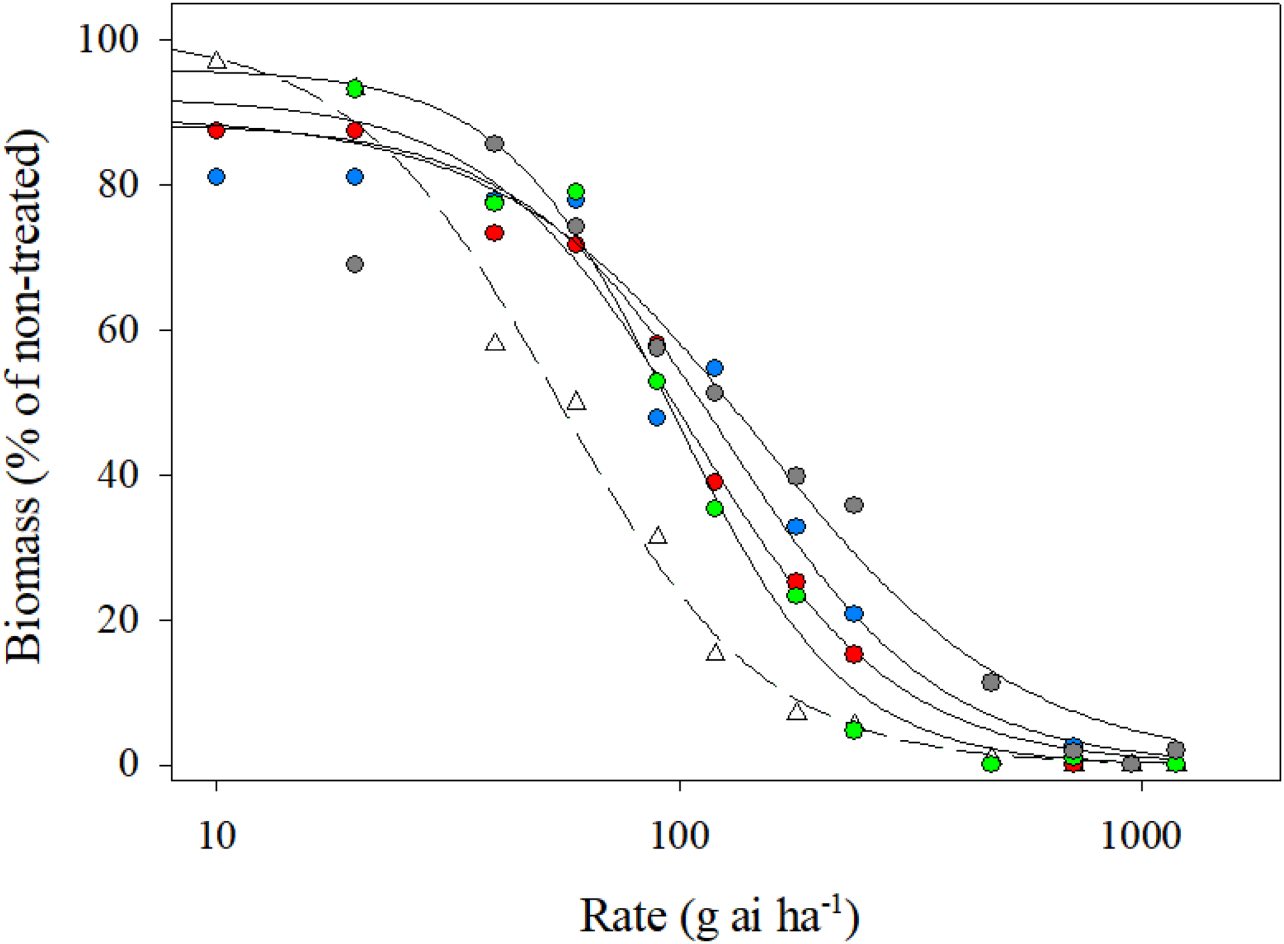
Dose–response curve fit to a three‐parameter logistic regression model for biomass of the *Amaranthus palmeri* populations (putative resistant: Anson County; susceptible: Johnston County) treated with glufosinate. Error bars were removed for clarity. Legend: Anson County: filled circle [A1: blue; A2: red; A3: green; A4: gray]; Johnston County: open triangle.

*Biomass*. The GR_50_ values were 109, 96, 94, 128 g ai ha^−1^ for the A1, A2, A3, and A4 accession, respectively (Table 3). The glufosinate GR_50_ value was 55 g ai ha^−1^ for the Johnston County population (Table 3). The different GR50 values further suggest differential susceptibility between the *A. palmeri* populations (Figure 3; Table 3) (Burgos 2015). Similar to the survival results, the A4 accession exhibited the least biomass reduction compared to the other accessions (Figures 2, 3; Tables 2, 3).

**TABLE 3:** Parameter estimates from the three‐parameter logistic regression for plant biomass of the putative glufosinate‐resistant Anson County *Amaranthus palmeri* accessions treated with glufosinate compared to the glufosinate‐susceptible Johnston County population.

Population	*a*	*x*0	*b*	*r* ^2^	GR_50_	GR_90_
Johnston county	101.0	54.7	1.9	0.99	55	168
A1	92.6	119.9	1.8	0.97	109	396
A2	94.7	102.3	1.9	0.99	96	322
A3	96.0	98.2	2.5	0.99	94	243
A4	89.6	149.6	1.5	0.96	128	556

*Note*: *a* is the upper asymptote, *x*0 equals the GR_50_, and *b* is the slope at *x*0.

Abbreviations: GR_50_, lethal dose to control 50% of the population; GR_90_, lethal dose to control 90% of the population; R/S, resistance ratio [GR_50_ resistant accession GR_50_ susceptible population^−1^].

### 3.3 A4 plants dose–response compared to susceptible populations with different glufosinate exposure

**FIGURE 4:**
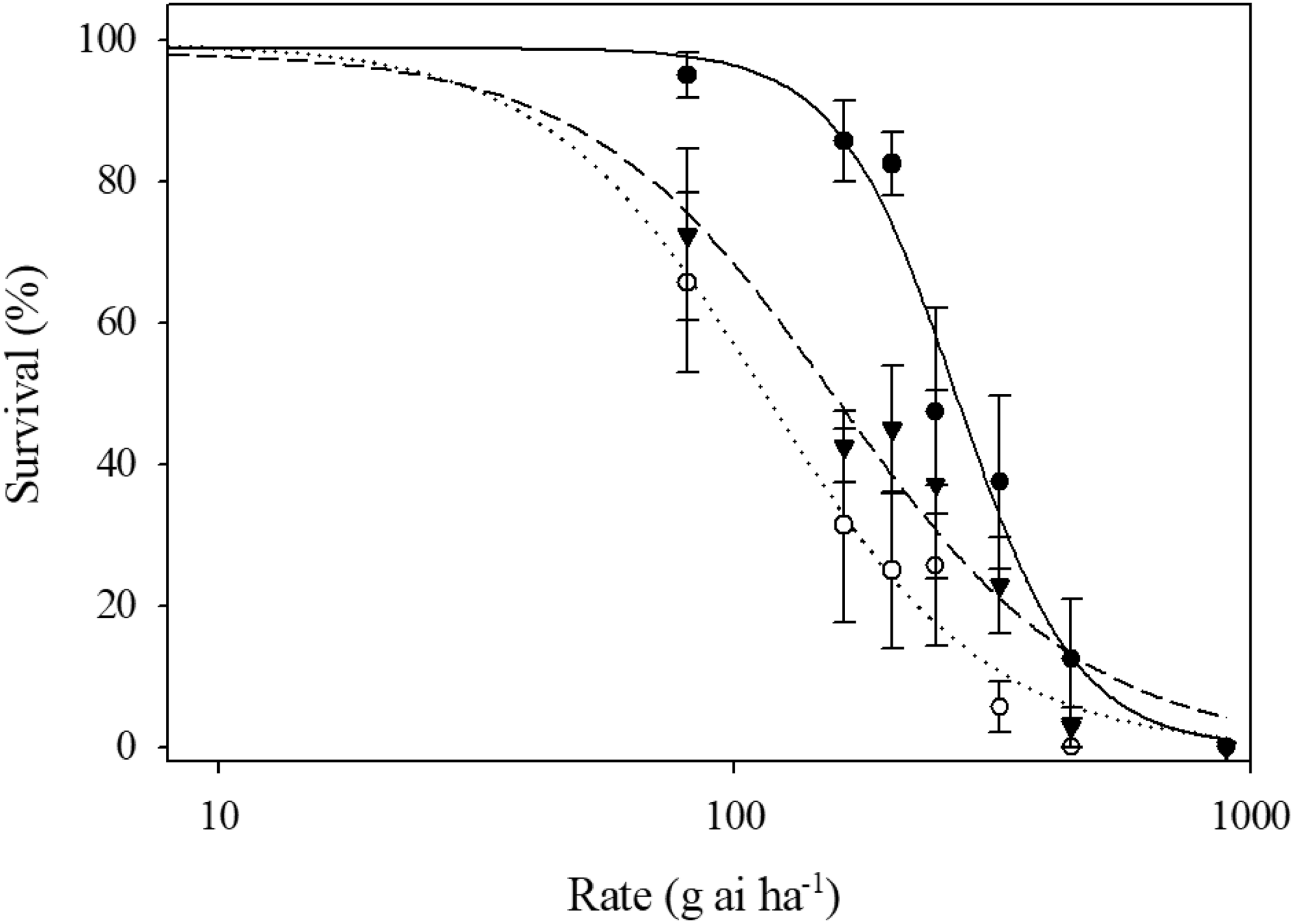
Dose–response curve fit to a three‐parameter logistic regression model for plant survival of the *Amaranthus palmeri* populations (putative resistant: Anson County; susceptible: Edgecombe and Lenoir County) treated with glufosinate. Error bars represent the standard error of the mean. Legend: Anson County: filled circle; Edgecombe County: open circle; Lenoir County: triangle.

The LD_50_ values were 267, 158, and 118 g ai ha^−1^ for the A4, Edgecombe, and Lenoir County populations, respectively (Figure 4; Table 4). While the LD_50_ values were dissimilar between the two dose–response experiments, the trend was consistent with the values of A4 being the highest. While only 10% of the A4 and Edgecombe County populations survived 450 g ai ha^−1^ glufosinate, similar research has demonstrated that this glufosinate rate has previously completely controlled susceptible populations in North Carolina (Mahoney et al., 2020; Poirier et al., 2014). This result suggests that the Anson County population is consistently surviving lethal glufosinate rates similarly to previously confirmed glufosinate‐resistant *A. palmeri* populations.

**TABLE 4:** Parameter estimates from the three‐parameter logistic regression for plant survival of the *Amaranthus palmeri* populations (putative resistant: Anson County; susceptible: Edgecombe and Lenoir County) treated with glufosinate.

Population	Regression parameters^a^				LD_50_	LD_90_
	*a*	*x*0	*b*	*r* ^*2*^		
Anson county	98.8	270.5	3.7	0.97	267	485
Edgecombe county	98.5	157.8	1.8	0.97	158	538
Lenoir county	99.6	115	2	0.98	114	337

Abbreviations: LD_50_, lethal dose (g ai ha^−1^) to control 50% of the population; LD_90_, lethal dose (g ai ha^−1^) to control 90% of the population.

^a^ *a* is the upper asymptote, *x*0 equals the LD_50_, and *b* is the slope at *x*0.

### 3.4 Response of F_1_ genotypes from the biparental crosses to glufosinate

**FIGURE 5:**
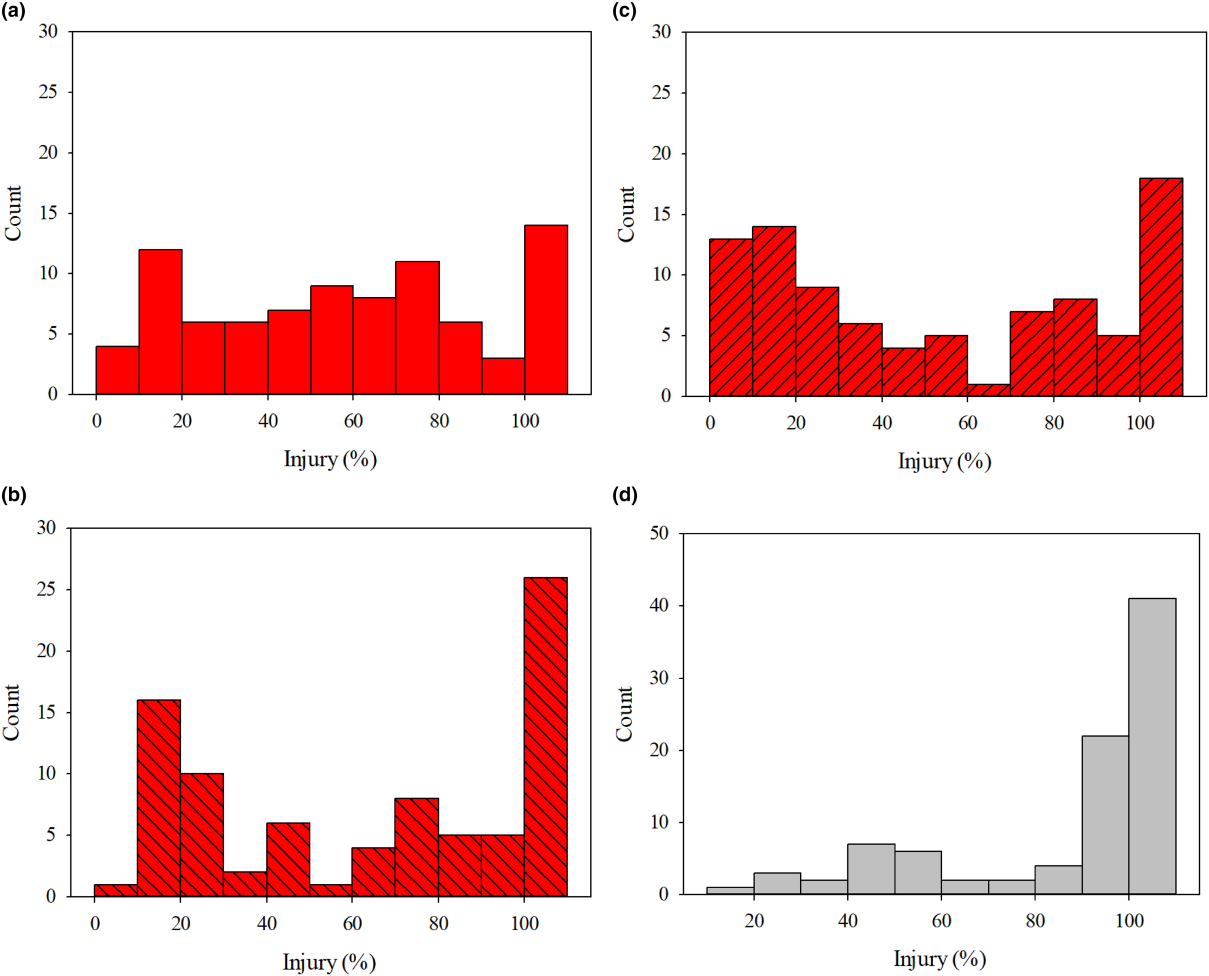
Distribution of injury across genotypes from the biparental crosses (a: resistant male surviving 450 g glufosinate ha^−1^ [RM] × resistant female surviving 267 g glufosinate ha^−1^ [RF_1_]; b: RM × susceptible female [SF]; c: Susceptible male [SM] × resistant female surviving 450 g glufosinate ha^−1^ [RF_2_]; d: SF × SM) when treated with glufosinate (267 g ai ha^−1^).

*Injury*. Environment (*p* = .002), biparental cross (*p* < .0001), and genotype (nested within biparental cross) (*p* = .0002) were significant main effects but the interactions were not (*p* = .08); thus, injury data were averaged over environment but analyzed separately for each biparental cross. Average injury for glufosinate‐treated plants was 55, 50, and 61% for the RM × RF_1_, SM × RF_2_ and RM × SF cross, respectively (Figure 5). The injury of crosses containing an Anson County parent followed variable distributions suggesting that response is quantitative (Figure 5). The average injury for plants from the SM × SF cross was 84%; approximately 20%–30% higher than the genotypes from crosses that included a parent plant from Anson County (Figure 4). The injury distribution exhibited by the plants from the SF × SM cross was severely skewed left reflecting that many of the treated plants exhibited high injury as expected from susceptible plants (Figure 5).

*Survival*. Environment (*p* = 0.16) was not a significant effect, while biparental cross (*p* < 0.0001) and genotype (*p* = 0.02) were significant main effects on clone survival. However, the interactions between main effects were not significant (*p* = 0.38); thus, survival data were averaged over environments and analyzed separately for each biparental cross. The average survival for glufosinate‐treated plants was 80, 80, and 68% for the RM × RF_1_, SM × RF_2_, and RM × SF crosses, respectively. The average survival of plants from the SM × SF cross was 35%; approximately 30%–45% lower than the crosses that included a parent plant from Anson County. The survival distribution exhibited by the plants from the SF × SM cross was severely skewed right elucidating that the plants were still exhibiting susceptibility (data not shown).

*Chi‐square goodness‐of‐fit*. The range of injury responses indicates an oligogenic trait/response is present. The RM × SF and SM × RF_2_ cross were best described with a heterozygous two loci with incomplete dominance model compared to the RM × RF_1_ cross that was best described with a heterozygous single locus with incomplete dominance model (Table 5). The SM × SF cross survival never fit any of the tested loci models, suggesting that the Lenoir County plants were indeed glufosinate‐susceptible (data not shown). The odds ratios of survival were 1.9 for both the RM × SF and RM × RF_1_ crosses compared to the SM × RF_2_ cross, which suggests that some glufosinate resistance gene(s) may be maternally inherited (Jasieniuk et al., 1996; Kohlhase et al., 2018). Noguera et al. (2022) found the chloroplast GS isoform was overamplified in a glufosinate‐resistant *A. palmeri* population from Missouri, which provided evidence that there was a resistance mechanism that could be maternally inherited (Reboud & Zeyl, 1994). Based on injury, glufosinate resistance was quantified as low to moderately heritable on a per plot basis (*H* ^*2*^ = 0.17) and a per entry basis (*H* ^*2*^ = 0.54), respectively. Comparatively, glufosinate resistance was quantified as low to highly heritable based on survival on a per plot (*H* ^*2*^ = 0.21) and per entry basis (*H* ^*2*^ = 0.71), respectively. These results indicated that glufosinate resistance from the Anson County population was heritable and likely an oligogenic trait with incomplete dominance.

**TABLE 5:** Chi‐square analyses for goodness‐of‐fit (GOF) of the number of loci contributing to the mechanism of glufosinate resistance in the F_1_ Anson County *Amaranthus palmeri* plants treated with glufosinate (267 g ai ha^−1^) 21 days after treatment.

Assumptions	Expected ratio^a^	Cross	Observed ratio	χ^2^	*p*
Single loci; homozygous parents	(0:1)	RM × RF_1_	(14:72)	>100	<0.0001
	(0:1)	RM × SF	(27:57)	>100	<0.0001
	(0:1)	SM × RF_2_	(18:72)	>100	<0.0001
Single loci; heterozygous allele parent	(1:3)	RM × RF_1_	(14:72)	11.3	0.0008
	(1:1)	RM × SF	(27:57)	10.7	0.0011
	(1:1)	SM × RF_2_	(18:72)	32.4	<0.0001
Two loci; homozygous allele parents	(0:1)	RM × RF_1_	(14:72)	>100	<0.0001
	(0:1)	RM × SF	(27:57)	>100	<0.0001
	(0:1)	SM × RF_2_	(18:72)	>100	<0.0001
Two loci; one homozygous and one heterozygous allele	(1:3)	RM × RF_1_	(14:72)	11.3	0.0008
	(1:1)	RM × SF	(27:57)	0.05	0.8167
	(1:1)	SM × RF_2_	(18:72)	7.2	0.0073
Two loci: two heterozygous	(6:10)	RM × RF_1_	(14:72)	16.5	<0.0001
	(3:1)	RM × SF	(27:57)	45.1	<0.0001
	(3:1)	SM × RF_2_	(18:72)	88.3	<0.0001
Single locus; incomplete dominance alleles from heterozygous parent	(1:2:1)	RM × RF_1_	(20:52:14)	4.6	0.1
	(3:0:1)	RM × SF	(17:40:27)	>100	<0.0001
	(3:0:1)	SM × RF_2_	(20:52:18)	>100	<0.0001
Two loci; incomplete dominance alleles from heterozygous parent	(8:7:1)	RM × RF_1_	(20:52:14)	24	<0.0001
	(1:2:1)	RM × SF	(17:40:27)	2.3	0.32
	(1:2:1)	SM × RF_2_	(20:52:18)	2.7	0.26

*Note*: Two separate analyses were conducted on the assumptions if the parent plants were homo‐ or heterozygous for glufosinate resistance.

Abbreviations: RM, resistant male surviving 450 g glufosinate ha^−1^; RF_1_, resistant female surviving 267 g glufosinate ha^−1^; RF_2_, resistant female surviving 450 g glufosinate ha^−1^; SF, non‐treated susceptible female; SM, non‐treated susceptible male.

^a^ Expected ratios were based off of survival (death: survival) and injury (intermediate resistant: >70%–90%; resistant: <70%; susceptible: >90%).

## 4 DISCUSSION

Noguera et al. (2022) and Priess et al. (2022) used glufosinate‐susceptible *A. palmeri* accessions collected in 2001 with limited exposure to herbicides; the tested glufosinate‐susceptible populations used in the present experiments were collected relatively recently (2013, 2019, and 2020) and were subjected to intensive herbicide exposure at the research stations. The glufosinate‐susceptible plant lines used in each experiment exhibited very different LD_50_ values (Arkansas: 40–63 g ai ha^−1^; North Carolina: 114–158 g ai ha^−1^). However, Anson County accessions exhibited a similar LD_50_ (210–316 g ha^−1^) value as two of the glufosinate‐resistant accessions from Arkansas (214 g ai ha^−1^) and the accession from Missouri (256 g ai ha^−1^). This result suggests that the Anson County population is consistently surviving lethal glufosinate rates similarly to previously confirmed glufosinate‐resistant *A. palmeri* populations. The LD_50_ of the Anson County population in the glasshouse is a historically efficacious rate in the field on similar sized *Amaranthus* plants, which further demonstrates that herbicide susceptibility within a genus/species can change spatially and temporally (Beyers et al., 2002; Corbett et al., 2004; Culpepper et al., 2000; Jones, Leon, & Everman, 2022). The difference in LD_50_ for the Anson County population may be exaggerated if compared to historic accessions as herbicide susceptibility can change spatially and temporally (Heap, personal communication) (Mahoney et al., 2020; Owen et al., 2014). The observed difference in susceptibility from the dose–response experiments was parallel to when a farmer would notice infestations of herbicide‐resistant weeds with 2‐ to 4‐fold differences in susceptibility (Gressel & Segel, 1990; Squires et al., 2021). The differential LD_50_ values between the populations that had different exposure times to glufosinate (low exposure: Lenoir County [114 g ai ha^−1^]; high exposure: Edgecombe County [158 g ai ha^−1^]) also provides evidence that recurrent use of glufosinate can cause a shift in susceptibility leading to the evolution of resistance.

Other weeds have polygenic mechanisms that facilitate glufosinate resistance (Brunharo et al., 2019). Although Carvalho‐Moore et al. (2022) found glufosinate resistance in *A. palmeri* was endowed by *GS* amplification and overexpression, previous research investigating glyphosate‐resistant *A. palmeri* (resistance endowed by amplification and overexpression of the *ESPS*) found that the responses were not best fit with a single gene model (Giacomini et al., 2013; Mohseni‐Moghadam et al., 2013). Salas‐Perez et al. (2018) found that more genes were expressed in an *A. palmeri* population that exhibited reduced susceptibility to glufosinate compared to susceptible populations, which follows the polygenic resistance mechanism found in the present research. The results of the presented research also suggest that glufosinate resistance genes can be transferred in the pollen; thus, the Anson County population could have intercepted pollen from a field under intensive glufosinate selection in proximity (Liu et al., 2012; Sosnoskie et al., 2012). However, Noguera et al. (2022) found the chloroplast GS isoform was overamplified in a glufosinate‐resistant *A. palmeri* population from Missouri, which provided evidence that there was a resistance mechanism that could be maternally inherited (Reboud & Zeyl, 1994). While a more elaborate crossing design (i.e., more genotypes/crosses and glufosinate rates tested) could reflect a more precise number of loci responsible for the mechanism(s) of resistance and the influence of maternally inherited gene(s) conferring variation in phenotype, the presented results provide an efficient approach of determining the inheritance and number of loci involved with glufosinate resistance in the Anson County population (Gressel, 2009; Kohlhase et al., 2018; Liu et al., 2019). Future research should focus on determining the exact mechanism(s) of resistance in the Anson County population.

The Anson County *A. palmeri* population has evolved resistance to glufosinate and the mechanism of resistance is oligogenic with a possible maternal effect (e.g., a plastid gene). Currently, glufosinate resistance is isolated in the Midsouth and Southeast United States, where glufosinate is applied extensively and intensively (Jones, Cahoon, et al., 2022; Riar et al., 2013). Although glufosinate is efficacious on most weeds elsewhere, continued sole reliance will select for more glufosinate‐resistant weeds (Owen & Zelaya, 2005; Schwartz‐Lazaro et al., 2018; Shergill et al., 2018; Shyam et al., 2021). Glufosinate resistance will inevitably increase the complexity of weed control in North Carolina; *Amaranthus palmeri* not controlled with glufosinate has been reported throughout North Carolina crops (Jones, Cahoon, et al., 2022). In tandem, approximately half of North Carolina farmers are using glufosinate to control *A. palmeri* and using the herbicide at multiple application timings (Jones, Cahoon, et al., 2022). *Amaranthus palmeri* populations should be collected from crop fields across North Carolina and screened to determine if there are more glufosinate‐resistant populations and the distribution of these populations.

## FUNDING INFORMATION

Project funding was provided by the North Carolina Soybean Producers association.

## CONFLICT OF INTEREST STATEMENT

The authors declare no conflict of interest.

## Supporting information


**Figure S1.** Illustration of the breeding pairs for the putative glufosinate‐resistant Anson County and ‐susceptible Lenoir County *Amaranthus palmeri* populations. Crosses were made from an accession (A4) from Anson County that exhibited the least susceptibility to glufosinate. The glufosinate rates in parentheses represents the rate that the plant survived in a dose–response assay conducted under glasshouse conditions.

## Data Availability

The manuscript contains summary data of all conducted experiments. The corresponding author will provide the original data upon request.
