# Supplementary material for: Confirmation and inheritance of glufosinate resistance in an Amaranthus palmeri population from North Carolina
Source: Plant Environ Interact. 2024 Jun 25;5(3):e10154. doi: 10.1002/pei3.10154 (PMC11199332; doi:10.1002/pei3.10154)
Supplement: Supplementary file 1 — Figure S1. Illustration of the breeding pairs for the putative glufosinate‐resistant Anson County and ‐susceptible Lenoir County Amaranthus palmeri populations. Crosses were made from an accession (A4) from Anson County that exhibited the least susceptibility to glufosinate. The glufosinate rates in parentheses represents the rate that the plant survived in a dose–response assay conducted under glasshouse conditions. [file PEI3-5-e10154-s001.docx]

**Supplemental figures**

**
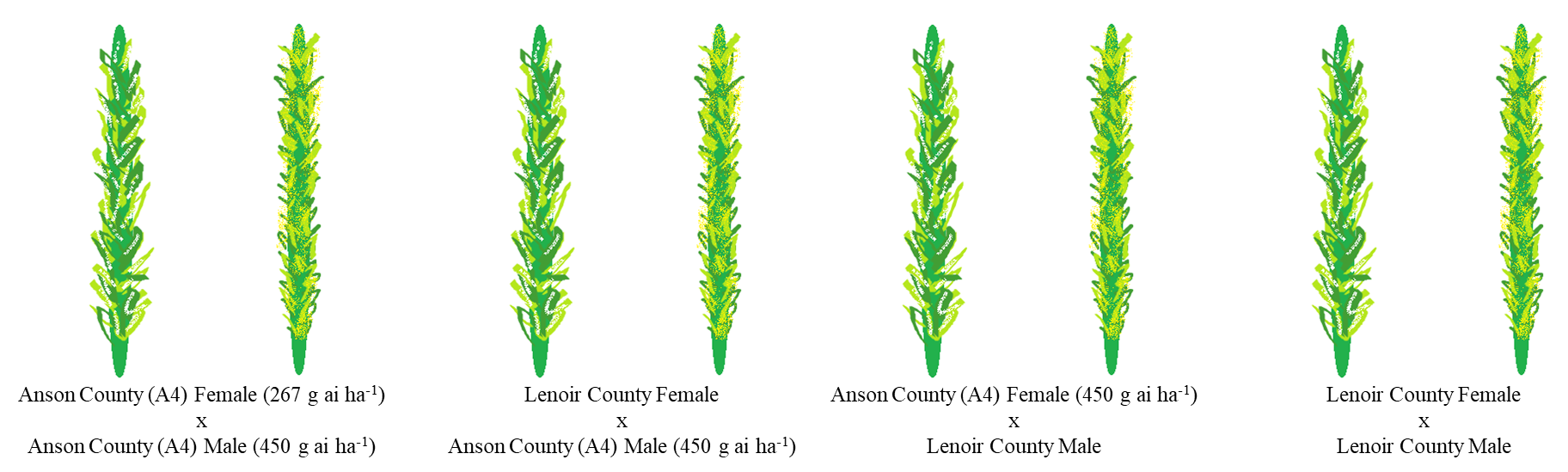
**

Supplemental figure 1. Illustration of the breeding pairs for the putative glufosinate-resistant Anson County and -susceptible Lenoir County *Amaranthus palmeri* populations. Crosses were made from an accession (A4) from Anson County that exhibited the least susceptibility to glufosinate. The glufosinate rates in parentheses represents the rate that the plant survived in a dose-response assay conducted under glasshouse conditions.
